# Comparison of repair of peripheral nerve transection in predegenerated muscle with and without a vein graft

**DOI:** 10.1186/s12883-016-0768-z

**Published:** 2016-11-22

**Authors:** Jamshid Mohammadi, Hamdollah Delaviz, Bahram Mohammadi, Hamoun Delaviz, Parastou Rad

**Affiliations:** 1 Medicinal Plants Research Centre, Faculty of Medicine, Yasuj University of Medical Sciences, Yasuj, Iran; 2 Cellular and Molecular Research Center, Faculty of Medicine, Yasuj University of Medical Sciences, P.o.Box: 7591994799, Yasuj, Iran; 3 Department of Pediatrics, Yasuj University of Medical Sciences, Yasuj, Iran; 4 The University of Toledo College of Medicine and Life Sciences, Toledo, OH USA; 5 Department of Midwifery, Yasuj University of Medical, Yasuj, Iran

**Keywords:** Vein, Conduit, Predegenerated, Muscle, Nerve, Repair

## Abstract

**Background:**

Despite substantial research into the topic and valiant surgical efforts, reconstruction of peripheral nerve injury remains a challenging surgery. This study was conducted to evaluate the effectiveness of axonal regeneration of a transected sciatic nerve through a vein conduit containing degenerated skeletal muscle compared with axonal regeneration in a transected sciatic nerve through degenerated skeletal muscle alone.

**Methods:**

In two of the three experimental rat groups, 10 mm of the left sciatic nerve was transected and removed. The proximal and distal ends of the transected sciatic nerve were then approximated and surrounded with either (a) a degenerated skeletal muscle graft; or (b) a graft containing both degenerated skeletal muscle and vein. In the group receiving the combined vein and skeletal muscle graft, the vein walls were subsequently sutured to the proximal and distal nerve stump epineurium. Sciatic functional index (SFI) was used for assessment of functional recovery. Tracing study and histological procedures were used to assess axonal regeneration.

**Results:**

At 60 days, the gait functional recovery as well as the mean number of myelinated axons in the middle and distal parts of the sciatic nerve significantly increased in the group with the vein graft compared to rats with only the muscular graft (*P* < 0.05). Mean diameter of myelinated nerve fiber of the distal sciatic nerve was also improved with the vein graft compared to the muscle graft alone (*P* < 0.05). The mean number of DiI-labeled motor neurons in the L4-L5 spinal segment increased in the vein with muscle group but was not significantly different between the two groups.

**Conclusions:**

These findings demonstrated that a graft consisting of not only predegenerated muscle, but also predegenerated muscle with vein more effectively supported nerve regeneration, thus promoting functional recovery after sciatic nerve injury in rats.

## Background

Traumatic peripheral nerve injury is common and many of these injuries lead to permanent disability and neuralgia. In spite of novel strategies to help bridge a peripheral nerve defect, typical nerve regeneration yields lackluster results far from original functional ability [1]. Epineurial repair is the most appropriate surgical procedure when the nerve is transected with a sharp object or when there is a small gap between the nerve endings [2]. But when the distance between the ends of the nerve defect is significant, repairing a transected nerve by the surgeon is very difficult or impossible without a graft or conduit tube. Therefore, search for a suitable guide channel which provides a microenvironment conducive for nutritional support and axonal growth in the gap has been of particular importance [3].

Functional recovery largely depends on axonal growth and guidance to the target organs. In various experimental studies, different methods which have been used include an autologous nerve graft, polypyrrole, artificial nerve conduit, vein and muscular graft [4–8]. It has been shown that use of a vein as a conduit bridge for a nerve gap less than 3 cm may support axonal regeneration [4]. Vein grafts for nerve loss have been used alone or with fresh skeletal muscle or predegenerated (freeze-thawed) skeletal muscle [9–11]. It appears that the nerve growth factor (NGF) from the wall of the vein may play a role in repair of the nerve injury [12]. However, collapse of vein is an issue that often occurs for longer nerve defects; such vein collapse can be prevented by filling the vein with muscle fibers [4].

An alternative approach for nerve injury is muscle graft, as it has been documented that an epimysial “tube” of muscle tissue could guide the regeneration of the proximal axon into the distal nerve stump in cases of peripheral nerve defects [9]. Transplantation of autologous muscle for use in repair of a nerve defect is most appropriate with the use of acellular autograft muscle in order to create favorable conditions for axonal growth and to prevent an immune response. Also, basal laminae without cells have been shown to protect axonal regeneration in mice [9]. Probably there was structure similarities between the epimysium and connective tissue that surrounding nerve fibers and the epineurium was sutured to the epimysium of the muscle to improve the neurotized remnant rectus abdominis muscle in patients [13, 14]. The epimysium has a dense connective tissue layer, extracellular matrix with property of bionic repair and it was a suitable channel to support the new axonal regeneration [15]. The hollow conduit of epimysium was ideal and could be an alternative option for nerve defect repair in clinical practice [15].

To the best of our knowledge, there is no specific experimental study which focuses on comparison of a graft containing vein with predegenerated muscle versus a graft containing predegenerated muscle alone for repair of peripheral nerve defects. Thus, the authors found it necessary to conduct a comparative study to evaluate the effectiveness of sciatic nerve repair in an animal model using a vein graft filled with muscle and a muscle graft alone. Evaluation of nerve repair was based on functional recovery, histological study of the sciatic nerve repair in the transplanted area and of the motor neuron of the lumbar spinal cord, as well as via determination of the quantity of motor neurons with using of DiI-labeled fluorescence,

## Methods

### Animal’s groups and surgery procedures

Thirty-six mature female Wistar rats (200–220 g) were divided into three equal groups of twelve. All the rats were anesthetized with an intraperitoneal injection consisting of a combination of ketamine (80 mg/kg) and xylazine (10 mg/kg). For sciatic nerve transection, in the vein with muscle graft group and in the muscle graft alone group, through a lateral incision in the mid-thigh and via a gluteal musculature the sciatic nerve was exposed and 2-cm distal to the sciatic notch 10 mm of the sciatic nerve removed by sharp microscissors [16]. In the sham-operated animals, the sciatic nerve was exposed but was not transected.

In the group receiving the muscle graft alone, one cm of gluteus superficial muscle was removed from the right side-opposite the side of nerve transection. The resected gluteus muscle was denatured by submerging it in liquid nitrogen for 50 s. Then it was thawed in distilled water for 3 min [17]. Under an operating microscope, the proximal and distal of the epineurium nerve ends were sutured to the epimysium of the muscle with 10–0 nylon, creating an endomysial tube in which the proximal and distal ends of the sciatic nerve were separated by 10 mm [15].

In the group receiving a graft combining vein with muscle, the left external jugular vein was exposed and mobilized [18]. Fifteen mm of the vein was resected and the transected ends of the external jugular vein were ligated to prevent hemorrhage. Subsequently, the resected vein was washed with saline solution, and a longitudinal incision was then established in the vein. Three mm of both the proximal and distal sciatic nerve stumps were covered by the jugular vein graft, leaving almost 10 mm between the proximal and distal nerve stumps. The venous wall was sutured to the nerve epineurium using 11–0 atraumatic sutures (Ethicon EH 7438G, Ethicon, Norderstedt, Germany). Nine mm of the denatured muscle was placed inside the vein in parallel with intact gluteus superficial muscle fibers. The thickness of the muscle graft was determined according to the internal diameter of the vein. In the middle part of the length of the vein, the lips of the vein were sutured by 10–0 nylon sutures to maintain the muscle inside the vein. Muscle and skin planes were then sutured with a 4–0 nylon monofilament. Penicillin-G (0.35 ml/kg intramuscular) was administered as a prophylactic antibiotic on postoperative days 1, 2 and 3. All three experimental groups were maintained on a 12 h light/dark cycle with water and food provided liberally throughout the experiment period.

### Footprint recording and analysis

For evaluation of motor function, a wooden channel with dimensions of 20 × 30 × 70 cm was designed. An assessment was performed for each group (*n* = 12) one day before the surgery and at 7, 14, 21, 28, 35 and 60 days after sciatic nerve transection by analyzing the rats’ walking tracks [19]. The left and right hind feet of the rats were dipped in dilute red and blue ink, respectively, and the animals were allowed to walk through the wooden channel toward a darkened box. The channel floor was covered with a sheet of paper to record their footprints. The print length (PL), toe spread from the first to the fifth toe (TS), and intermediary toe spread (IT) from the second to the fourth toe were measured on the left or experimental side (EPL, ETS, and EIT) as well as on the right or normal side (NPL, NTS, and NIT). The sciatic functional index (SFI) was calculated according to the following formula [19]:

$$ \mathrm{S}\mathrm{F}\mathrm{I} = \hbox{--} 38.3\ \left[\left(\mathrm{E}\mathrm{P}\mathrm{L}\hbox{--} \mathrm{N}\mathrm{P}\mathrm{L}\right)/\mathrm{N}\mathrm{P}\mathrm{L}\right] + 109.5\ \left[\left(\mathrm{E}\mathrm{T}\mathrm{S}-\mathrm{N}\mathrm{T}\mathrm{S}\right)/\mathrm{N}\mathrm{T}\mathrm{S}\right] + 13.3\left[\left(\mathrm{E}\mathrm{I}\mathrm{T} - \mathrm{N}\mathrm{I}\mathrm{T}\right)/\mathrm{N}\mathrm{I}\mathrm{T}\right]-8.8 $$

A SFI of around zero indicates normal nerve function and a SFI of around −100 represents complete dysfunction.

### Tracing study

Two months after treatment, six animals from each group were anesthetized with an intraperitoneal injection of a mixture of ketamine (90 mg/kg) and xylazine (10 mg/kg). After general anesthesia, 40 μl of saturated DiI (1, 1-dioctadecyl-3, 3, 3, 3–tetramethylindocarbocyanin perchlorat) from Molecular Probes (Leiden, Netherlands; cat. No, D-282) in DMSO was injected at four points in the left gastrocnemius muscle [20]. Ten days later, the rats were deeply anesthetized with a double dose of ketamine (200 mg/kg) and xylazine (20 mg/kg) and perfused via a cardiac vessel with 0.9% heparin and with a fixative containing 4% paraformaldehyde and 1.25% glutaraldehyde in a 0.1 M/L sodium phosphate buffer (pH 7.2). Lumbar spinal segment L4-L6 was dissected out and placed in 30% sucrose overnight. Serial 50 μm-thick transverse sections of the spinal segment were made using a freezing microtome (leica cryostat, CM 3000). In each group, the labeled motor neurons were counted in the left anterior horn using fluorescent microscopy (Olympus Ax70).

### Histological investigation

The myelinated nerve fibers at the transplanted area and the morphology of the motor neurons of the anterior horn of left L4-L6 spinal cord were studied by light microscopy in the designated six rats from each group. In each of the 18 rats, a 15-mm long sciatic nerve segment of the sciatic nerve-which included the area of original transection as well as transplant tissue was transected and divided equally into three 5 mm proximal, middle and distal segments. Both the proximal and distal ends of the excised nerve have been marked. These transected parts plus the thin slices of the L4-L6 spinal cord from the six rats from each group were fixed for 2 h in 2.5% glutaraldehyde. Then, these nervous tissue specimens were washed in 0.1% cacodylate buffer and postfixed in 1% osmium tetroxide containing 0.8% potassium ferrocyanide and 5 nM calcium chloride in a 0.1 M cacodylate buffer for 90 min. The samples were then dehydrated through graded acetone solutions, infiltrated with Poly/Bed 812 resin (Polysciences, Inc.,Washington, PA) and polymerized at 65 °C for 60 h. Ultrathin sections (80–90 nm) were cut with an ultramicrotome (Leica ultracut UCT), dyed with uranyl acetate (1%) and lead citrate (0.1%) and examined by microscope (Olympus Ax70). Transverse semithin sections of the proximal, middle and distal segments sciatic nerve were examined. For each 5 mm segment, the number of axons per 5 area (1363 μm^2^) of each section were counted with using microscope (Olympus Ax70) and image analysis computer system (olysia). As described previously [21], the number of axon in each section were summed per rat to give the numbers of the 5 mm long segment of the each rat. Additionally, in every five cross sections of the 5 mm sciatic nerve segment myelinated nerve fiber diameter were measured.

### Data analysis

Data analysis was performed using one-way ANOVA followed by the Tukey's post-hoc test analysis (Prism 5; Graphpad Software Inc., San Diego, CA). The results were expressed as mean ± standard deviation. A p-value of less than 0.05 was considered to be statistically significant.

## Results

### Gait functional analysis via sciatic function index

**Fig. 1:**
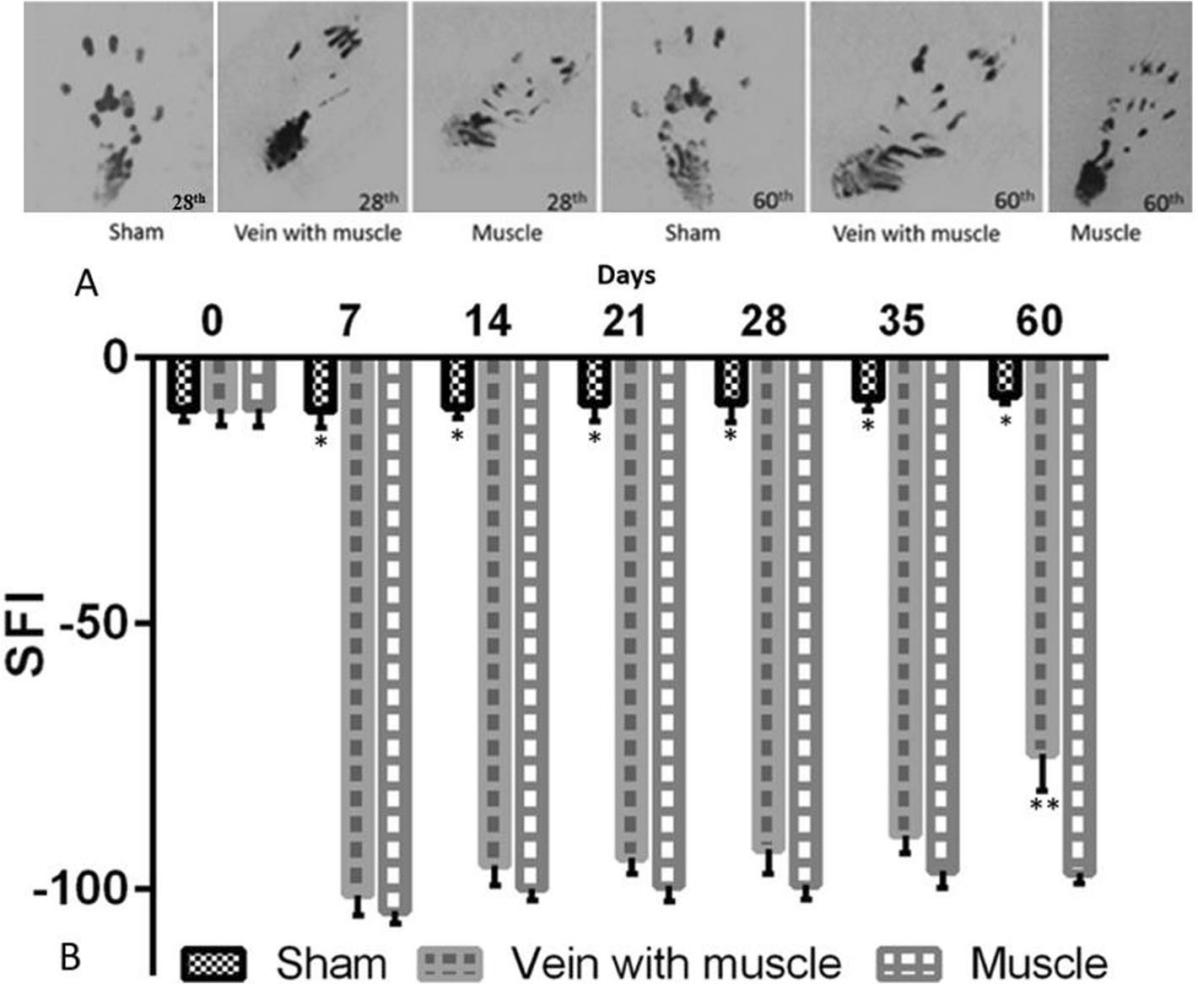
Vein with muscle group significantly improved the functional recovery of the injured sciatic nerve. Walking track analysis (**a**), of the rat prints on the injured left side at 28 and 60 days after treatment. Sciatic functional index (SFI) values are expressed as mean ± SD, n = 12, *p < 0.001 compared to the vein with muscle and the muscle only graft groups, **p < 0.05 compared to the muscle only graft group (**b**)

Photographs of the rat prints demonstrated the functional recovery improved from 28 toward 60 days post treatment in the both grafted groups (Fig. 1a). On the day before the surgery, the functional assessment based on SFI was carried out for each group. Moreover, no significant difference between groups and the result was reported. However, SFI was found to be greatly decreased at postoperative day seven in both the experiment group which received the vein with muscle graft and in the experimental group which received the muscle graft alone, especially compared to the sham group (*P* < 0.001), (Fig. 1b). Differences in mean SFI values were not significant between the group with the vein and muscle graft compared to the group with the muscle only graft from the first week postoperatively until the end of the fifth week. Yet 60 days after treatment, the gait functional recovery improved in the vein with muscle graft group (−74.6 ± 6.9) compared to the muscle only graft group (−96.9 ± 2.1) of rats (*P* < 0.05), (Fig. 1b).

### DiI- labeled motor neurons

**Fig. 2:**
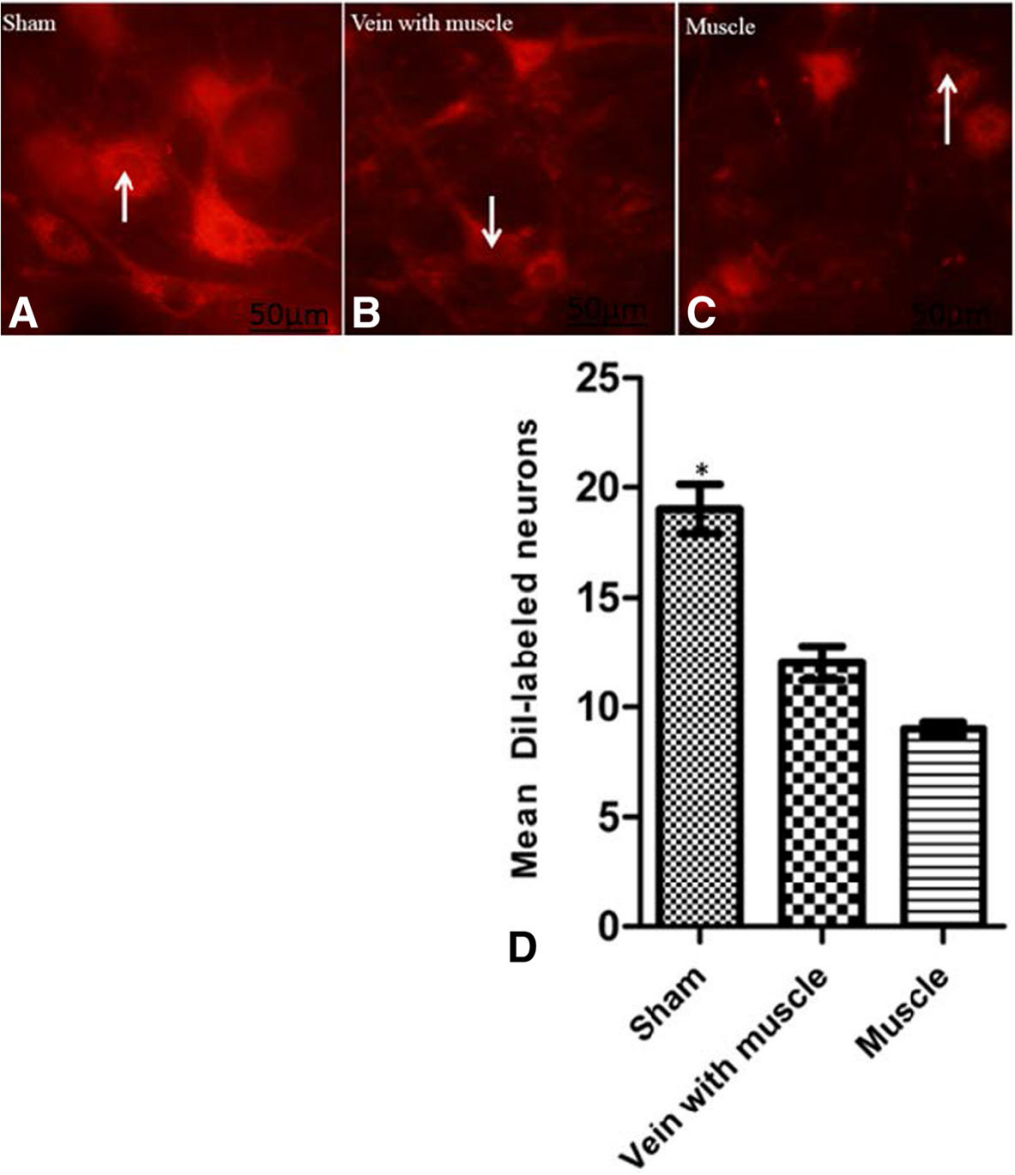
Vein with muscle has better outcome than muscle alone on axonal regeneration after nerve injury. DiI-labeled motor neurons in L4-L6 segments in different groups seventy days after treatment. The nucleus of the motor neuron appears darker than the cytoplasm (arrows, **a**, **b**, **c**). The DiI-labeled neurons values are expressed as mean ± SD, n = 6, *p < 0.01 compared to the vein with muscle and the muscle only graft groups (**d**). Scale bar: 50 μm (**a**, **b**, **c**), Magnification × 400 (**a**, **b**, **c**)

As shown in the Fig. 2a, the DiI tracers were retrogradely transported from the gastrocnemius muscles along axons to label spinal motor neurons seventy days after treatment. The existence of DiI retrograde labeling in some motor neurons of the spinal ventral horn indicated that the regenerating axons from the sciatic nerve had passed through the vein with muscle graft or muscle-only graft and eventually reached the target organ (Fig. 2b, c).

As expected the mean number of DiI labeled motor neurons in the sham group (19 ± 1.11) was significantly higher compared to the other two groups (*P* < 0.01). The mean number of the labeled motor neurons increased in the vein with muscle graft group (12 ± 0.8) compared to the muscle-only graft group (9 ± 0.3), but the difference did not reach statistical significance (*p* > 0.05), (Fig. 2d).

### Qualitative and quantitative histological changes

**Fig. 3:**
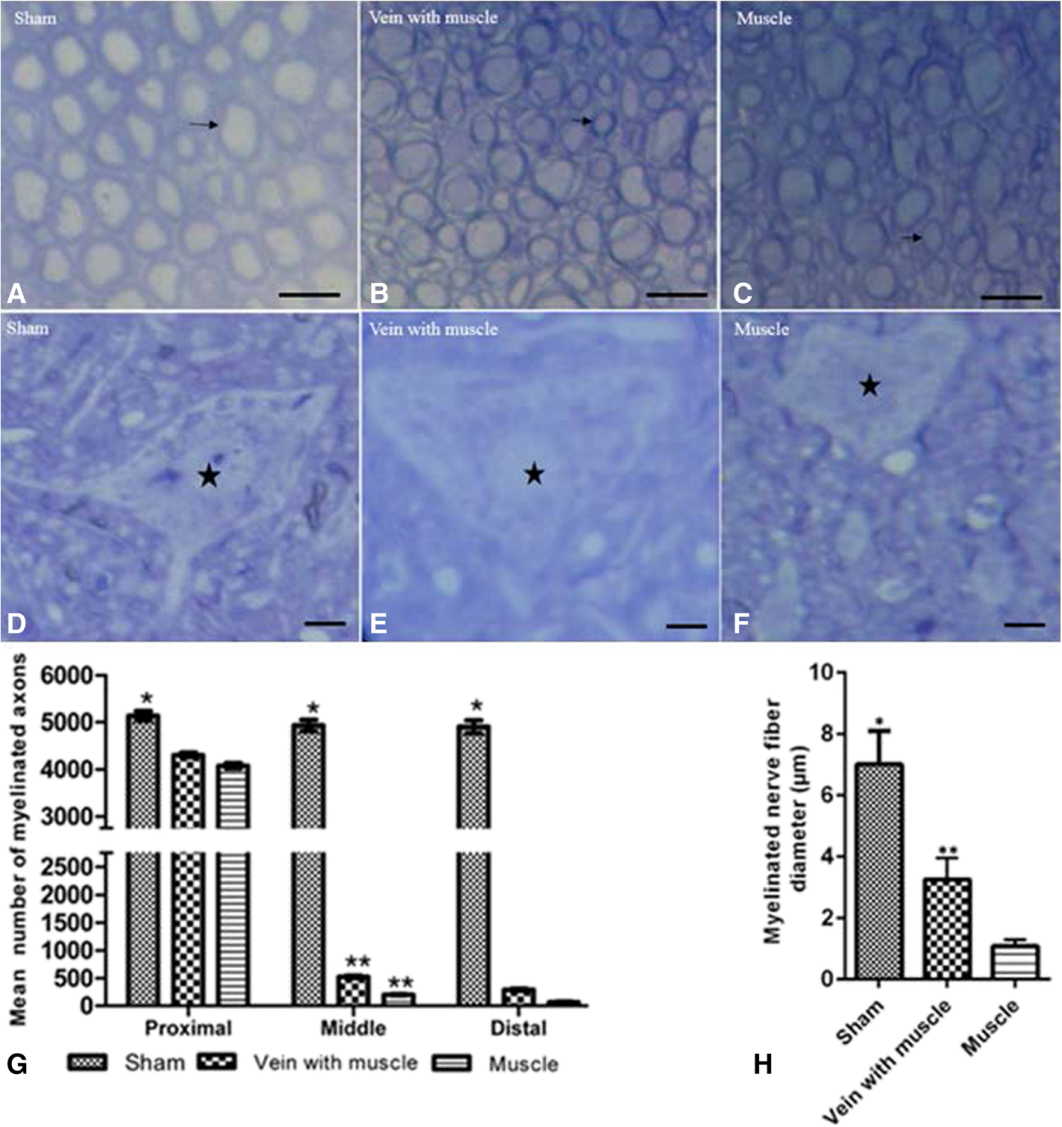
Average diameter and axon number significantly increased in the vein with muscle group. Micrographs of the transverse semi-thin sections of the distal segment of the sciatic nerve (**a**, **b**, **c**) and L4-L6 lumbar spinal neuron (**d**, **e**, **f**). Thick and intact myelin axons (arrow, **a**) with healthy multipolar neuron (**d**) are seen in the sham group. Myelinated axons in different diameter (**b**) with swollen of nucleus without nucleoli (**e**) are seen in the vein with muscle graft group. Axons are much thinner (arrow, **c**), degenerating nerve fibers (double arrow, **c**) peripheral displacement of the nucleus, disappearance of the nucleolus and loss of nissl body are seen (**f**) in the muscle group. *p < 0.001 compared to the vein with muscle and the muscle groups (**g**, **h**). *p < 0.05 compared to the muscle group (**g**, **h**). All images were stained with methylene blue; Scale Bar: A, B and C, 15 μm; D, E and F, 25 μm, *n* = 6

As shown in the Fig. 3, semi-thin transverse sections of the 5 mm segments of the distal sciatic nerve in the sham group, a high density of intact axons was seen (Fig. 3a). In the other two groups which received the sciatic nerve transection, the axonal regeneration was surrounded by thin and sparse myelin sheath 60 days after repair (Fig. 3b, c). The myelin sheath thickness in the vein with muscle graft group was observed to be greater than the muscle graft alone group (Fig. 3b, c). The irregular and degenerated axons were also seen in the muscle graft alone group (Fig. 3c).

Semi-thin sections of motor neuron of L4-L6 lumbar spinal cord in the sham group rats demonstrated a multipolar neuron with nissl substance distributed throughout the perikaryon (Fig. 3d). The nucleus was large, round and euchromatic with a single defined nucleolus (Fig. 3d). In the vein with muscle graft group, the perikaryon observed to be triangular, the nucleus membrane observed clear and located nearly in the center of the nerve cell (Fig. 3e). The chromatin appeared more dispersed and the nucleolus was not clear in the nucleus (Fig. 3e). Displacement of the nucleus, disruption of the nuclear membrane and basophilic nissl substance was condense and near the nucleus in the muscle graft alone (Fig. 3f).

Tukey's post-hoc test revealed there was no statistically significant difference in the mean nerve fibers between vein with muscle graft group compared to the muscle graft alone group regarding the sections taken from the proximal part of the sciatic nerve (*p* > 0.05). So, the distal and middle segments of the sciatic nerve was evaluated.

There was a statistically significant difference in the average number of myelinated nerve fibers in the middle and distal sections of the repaired sciatic nerve between the group with the vein and muscle graft compared group with the muscle only graft (*p* < 0.05) (Fig. 3g). As shown in Fig. 3h, similarly, Tukey's post-hoc test revealed a significant difference in mean diameter of myelinated nerve fiber of the distal segment of the sciatic nerve between the vein with muscle graft group (3.24 ± 0.37) compared to the muscle only graft group (1.09 ± 0.2), (*P* < 0.05). Semi-thin transverse sections showed that the diameter of myelinated nerves fiber in the sham group was greater than in the other two experimental groups (*P* < 0.001), (Fig. 3a, h).

## Discussion

Although different methods exist to repair peripheral nerve gaps, functional recovery is far from optimal [22, 23]. So the management of the nerve repair or choice of method remains a major challenge for the surgical team. However, non-neural autologous grafts involving vein, muscle and artery are important not only for experimental research but also for clinical practice. Since these kinds of grafts are more accessible, far cheaper, easy to perform and have the potential of facilitating nerve repair [24–26].

Results of the present study indicated that grafts involving vein and predegenerated muscle and grafts involving predegenerated muscle alone support effective repair of peripheral nerve defects of 10 mm in rats. In the present study, the number of DiI-labeled neurons in the vein with muscle graft increased 15.79% compared to the muscle graft alone if we considered the sham group 100%. It has been documented that small changes in axonal regeneration could significantly have an impact on the locomotor function [3].

The advantage of using a vein conduit for nerve defect less than 3 cm has been demonstrated in the literature [1, 4, 8]. Likewise, the beneficial effect of using a fresh or predegenerated muscular graft has been reported by many authors [9, 10, 27]. Taking this idea one step further, our results indicate that a vein with muscle autograft creates a more favorable environment for nerve repair compared to a muscle-only autograft. There are many reasons that using a vein provides a lot of advantages for nerve repair, including neurotrophic factors such as NGF released from the endothelial layer of the vein [12]. Further, an autogenous vein grafts over end-to-end epineural suturing have chemical and mechanical assisted to made more regeneration axons and also reduced the epineural scarring (18). While the laminin and collagens in the vein wall provide a favorable internal environment for the regrowth of nerve fibers [28].

The results of the present study confirmed the Brunelli et al. study which described how using a graft containing vein with fresh skeletal muscle provided the better outcome than a graft containing muscle alone [29]. In the Brunelli et al. study, morphological analysis of the nerve graft in the transplanted area of the grafted group improved compared to the sham group which indicated with no difference between them after 6 months of treatment [29]. The better outcomes of Brunelli et al. study compared our result may be related to use of the fresh skeletal muscle with vein graft for 6 months for nerve repair in their study, while we used denatured skeletal muscle in the vein graft for 2 months. Denatured skeletal muscle may release more substances, impeding nerve fiber regeneration inside the vein [30]. For long nerve defects, muscle in veins may prevent collapse of the vein graft, thus helping guide axonal regeneration to the distal target [31]. A vein graft filled with muscle may also prevent adherence and fibrosis of the vein [32]. The basal lamina of skeletal muscle exhibits similarities with the endoneurial tube, allowing for effective results in nerve fiber regeneration [28].

In one study, the vein-adipose graft compared the vein-muscle graft in 1 cm-long defect of the median nerve demonstrated the adipose tissue remained in the vein after 6 months and worsened the nerve repair compared to the vein-muscle graft [33]. The basal lamina of the predegenerated skeletal muscle could build a network of directed tubes which act as conductor for axonal regeneration and organization [34]. Pretreatment of skeletal muscle leads to necrosis of different cells removed by macrophage cells and could help the regenerating axon to find a reduced amount of barrier [30]. Histological analysis of the nerve repair by vein with fresh skeletal muscle demonstrated the regenerating axon has an appropriate morphology of regeneration [14]. Both the predegenerated and fresh skeletal muscle are effective tissue-engineered for peripheral nerve repair and fresh muscle produce neurotrophic factors and stimulate the Schwann cell migration that promote axonal regeneration [35, 36]. Further, Geuna et al. have reported on the effectiveness of using grafts composed of fresh-muscle and vein for repairing long 55-mm nerve gaps in adult male rabbits [37]. Based on our knowledge, there are a few studies which have used denatured muscle to repair nerve defect in patients. In one study on 38 patients with leprosy the autografts of freeze-thawed skeletal muscle protected the median nerve from of the ulcers and blisters in the ten of eleven patients and sensory recovery was improved in 34 patients (89%) in hand and food after follow-up of 12.6 years [38]. The beneficial effect of using a vein filled with muscle to bridge nerve defects has been confirmed in clinical practice compared to patients with strictly autologous nerve grafts [10]. The clinical efficacy of a graft containing both muscle and vein have been reported in nerve defects up to 4 cm when involving nerves of the hand and up to 6 cm when involving nerves of the forearm [39].

## Conclusions

The results of the present study demonstrated that following nerve transection a graft containing both denatured muscle and vein compared to a graft containing denatured muscle alone resulted in better functional recovery and more effective nerve fiber regeneration. These findings suggest that a more rational framework for treatment of peripheral nerve injuries involves using an autologous graft containing vein filled with muscle.

## Abbreviations

EIT: Experimental intermediary toe
EPL: Experimental print length
ETS: Experimental toe spread
IT: Intermediary toe
NIT: Normal intermediary toe
NPL: Normal print length
NTS: Normal toe spread
PL: Print length
SFI: Sciatic function index
TS: Toe spread.
